# Effects of a Lysine-Involved Maillard Reaction on the Structure and In Vitro Activities of Polysaccharides from Longan Pulp

**DOI:** 10.3390/molecules24050972

**Published:** 2019-03-10

**Authors:** Yang Yi, Miao-Miao Han, Fei Huang, Li-Mei Wang, Ting Min, Hong-Xun Wang

**Affiliations:** 1 College of Food Science & Engineering, Wuhan Polytechnic University, Wuhan 430023, China; yiy86@whpu.edu.cn (Y.Y.); 18211673915@163.com (M.-M.H.); minting1323@163.com (T.M.); 2 Sericultural & Agri-food Research Institute, Guangdong Academy of Agricultural Sciences, Key Laboratory of Functional Foods, Ministry of Agriculture, Guangdong Key Laboratory of Agricultural Products Processing, Guangzhou 510610, China; hf1311@163.com; 3 College of Biology and Pharmaceutical Engineering, Wuhan Polytechnic University, Wuhan 430023, China; wanglimeiyx@163.com

**Keywords:** longan pulp, polysaccharide, Maillard reaction, modification, structure, bioactivity

## Abstract

The effects of amino acid-involved Maillard reactions (MRs) on the structure and activities of longan pulp polysaccharides (LPs), which were heteropolysaccharides mainly composed of glucose, galactose, mannose, rhamnose, glucuronic acid, ribose, and galacturonic acid, were investigated. The changes of browning degree and molecular weight (*M_w_*) distribution in the MR systems containing LPs and amino acids (lysine, proline, or glycine) indicated that lysine was more active in conjugating with LPs. The MR-modified LPs (MLPs) obtained via a 4 h MR between LPs and lysine showed obvious structural differences from LPs. Specifically, particle-like LPs contained 94% fractions with a *M_w_* less than 7.07 kDa, by contrast, network-like MLPs contained 45% fractions with a *M_w_* larger than 264.1 kDa. Moreover, MLPs showed stronger radical scavenging abilities and macrophage immunostimulating effects, but weaker cancer cell growth-inhibitory abilities. The results indicate that the amino acid-involved MR is a promising method to modify native polysaccharides for better biological properties.

## 1. Introduction

Longan (*Dimocarpus longan* Lour.) is a subtropical fruit commercially planted in China, Thailand, India, and Vietnam. Its pulp attracts great attentions due to desirable taste and nutrition. In traditional Chinese medicine, this pulp has been chronically used as a tonic to prevent and cure diseases, such as amnesia, insomnia, palpitation, and so on [1]. Pharmacological studies have confirmed that longan pulp has a variety of bioactivities, of which antioxidant, anticancer, and immunomodulatory activities have been mainly attributed to its polysaccharides [1,2,3,4]. Accordingly, longan pulp is an ideal additive for functional foods and an excellent candidate for modern medicine.

The biological activities of polysaccharides greatly depend on their molecular structures, which include a sugar unit and glycosidic bond in the main chain, the type and polymerization degree of the branch, and the molecular weight (*M_w_*), flexibility and conformation of the multichains [5,6]. To enhance the application performance of polysaccharides, structural modifications have been widely concerned with the pursuit for stronger or new bioactivities. Many methods, including sulfation, phosphorylation, carboxylic acid esterification, sulfonic acid esterification, carboxymethylation, and acid hydrolysis, have been applied to the functional improvement of polysaccharides. The Maillard reaction (MR), which occurs between the reactive carbonyl group of carbohydrates and the amino group of proteins (amino acids) under mild and safe conditions and requires no extraneous chemicals, is highly recommended as a natural, nontoxic, and effective method for macromolecular modification [7,8,9,10,11]. Until now, the structural modification of longan pulp polysaccharides, especially the MR-based modification, has been rarely reported.

Previous studies on the MR-based modification of macromolecules mainly focused on the changes of modified proteins on structure and functional properties [10,11]. As the macromolecular counterpart related to MR, polysaccharides have been poorly examined. Our previous work confirmed the MR between polysaccharides and proteins from longan pulp [4]. It also suggested the possibility of MR between longan polysaccharides and amino acids. Sun et al. [12] indicated that amino acid-involved MR significantly changed the structure and rheological property of *β*-glucans. Up to now, studies regarding the influences of amino acids on the structure and bioactivity of polysaccharides via the MR are few.

In the present study, the main fractions of longan pulp polysaccharides (LPs) were obtained through sequential technologies including water extraction, alcohol precipitation, and gel filtration chromatography. The MR characteristics of LPs with different amino acids were measured. The LP conjugates were further isolated to investigate the structural and functional changes compared with LPs. The comparison indicates the effects of amino acid-involved MR and identifies the feasibility of the MR-based modification.

## 2. Results

### 2.1. Purification of Longan Pulp Polysaccharides

**Figure 1:**
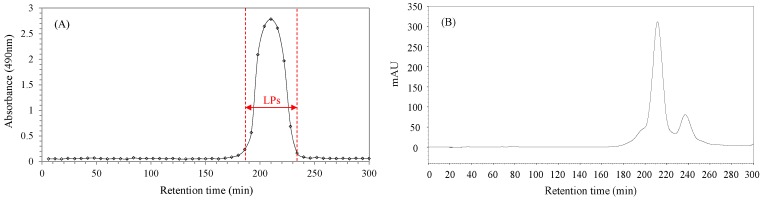
The size exclusion chromatography (SEC) chromatograms of longan pulp polysaccharides (LPs): (**A**) Detection by the phenol-sulphuric acid method (490 nm); (**B**) Detection by a UV detector (280 nm).

The *M_w_* of crude polysaccharides from longan pulp mainly ranged from 4.37 kDa to 91.8 kDa [13]. Accordingly, a HiPrep Sephacryl S-100 gel column designed for the isolation of macromolecules in the *M_w_* range of 1–100 kDa was selected for the purification. As seen in Figure 1A, there is only one relatively symmetrical peak in the size exclusion chromatography (SEC) chromatogram belonging to the main fractions (LPs). However, there are two protein peaks in the same range as seen in Figure 1B. These chromatograms suggest that LPs are composed of two sub-fractions containing bound or unbound proteins.

### 2.2. Physicochemical Changes during MR between LPs and Amino Acids

**Figure 2:**
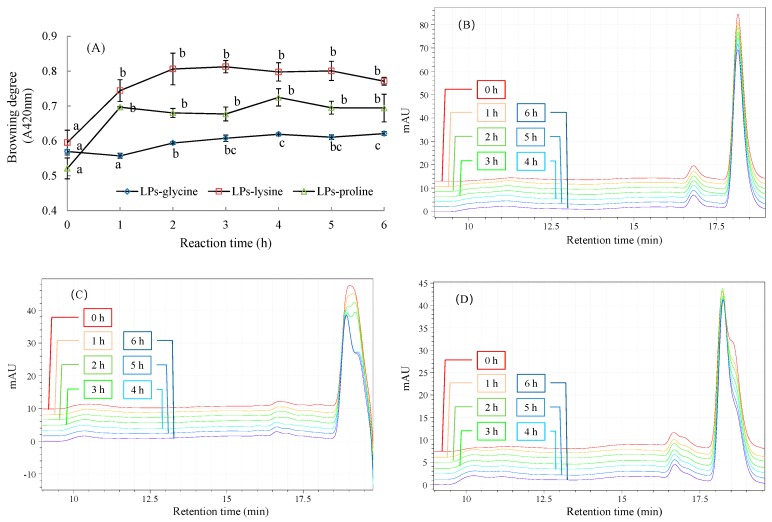
The physicochemical changes during the Maillard reaction (MR) between LPs and amino acids: (**A**) The change of browning degree; (**B**–**D**) the changes of molecular weight distribution in the LPs–glycine, LPs–lysine and LPs–proline reaction systems, respectively.

Browning is related to the formation of colored products in the advanced MR stages, which can be determined by the 420 nm absorbance [7]. The browning trends of the LPs–Lys and LPs–Pro systems were almost same (Figure 2A). Their browning degrees significantly increased at the beginning (*p* < 0.05), and then remained stable during the following 5 h reaction (*p* > 0.05). The browning of the LPs–Gly system, by contrast, was much weaker.

The effect of amino acid-involved MR on the *M_w_* of LPs was investigated by a HPSEC-RI method (Figure 2B–D). There are two main peaks in the chromatograms. The small one in the retention time range of 16.5–17.0 min belongs to the fraction of LPs with higher *M_w_*. The signals of another fraction and amino acid overlap are seen in the large peak ranging from 17.8 min to 18.7 min. The retention times in the three chromatograms are slightly different, which may be related to the *M_w_*-dependent performance of the SEC column (Ultrahydrogel 250, 7.8 mm × 300 mm, Waters). The shape change of the overlapped peaks indicates the reaction between the low M*_w_* fraction and amino acids. The overlapped peaks of the LPs–Gly system did not change during the 6 h reaction. By contrast, those of the LPs–Lys and LPs–Pro systems obviously changed. In detail, the right signal belonging to Lys or Pro gradually weakened with the increase of reaction time (Figure 2C,D), while the left signal increased without obvious change in retention time. The results imply that Lys and Pro gradually conjugate with the carbonyl groups of the LPs, and the grafted amino acids do not significantly change the *M_w_* of the LPs.

### 2.3. Chemical Compositions of LPs and MLPs

According to the characteristic of the MR with LPs, Lys was chosen to modify the LP structures, and MLPs were obtained after a 4 h reaction. LPs were mainly composed of polysaccharides and proteins with 25.92 μmol/g dry weight (DW) free amino groups (Table 1). By comparison, the weights of polysaccharides, proteins, and free amino groups in the MLPs were lower. The decreased weights of polysaccharides and proteins, which amounted to about 25.28 g/100 g DW, might be mainly related to the grafted Lys. We estimate that the grafted Lys accounted for approximately 25.28 g/100 g DW in the MLPs.

LPs and MLPs were both composed of mannose (Man), ribose (Rib), rhamnose (Rha), glucuronic acid (GlcA), galacturonic acid (GalA), glucose (Glc), and galactose (Gal), but their monosaccharide compositions were slightly different. The higher molar ratios of Man, Rha, GlcA, and Gal to Glc in MLPs might be related to the dominating consumption of Glc. The decreased molar ratios of Rib and GalA indicate their consumptions in the MR. Specifically, the significant decrease of Rib might be due to its higher reaction sensitivity compared with aldohexose [14].

### 2.4. Structural Features of LPs and MLPs

Compared with LPs, MLPs had a stronger absorbance at 260 nm due to the covalent binding of Lys to LPs via MR (UV–Vis spectra not shown). In addition, MLPs had no obvious absorption peak at 294 nm, which was derived from the uncolored compounds formed in the intermediate reaction stages [4]. These compounds might be removed by dialyzing against distilled water because of their relatively small molecular weights. 

**Figure 3:**
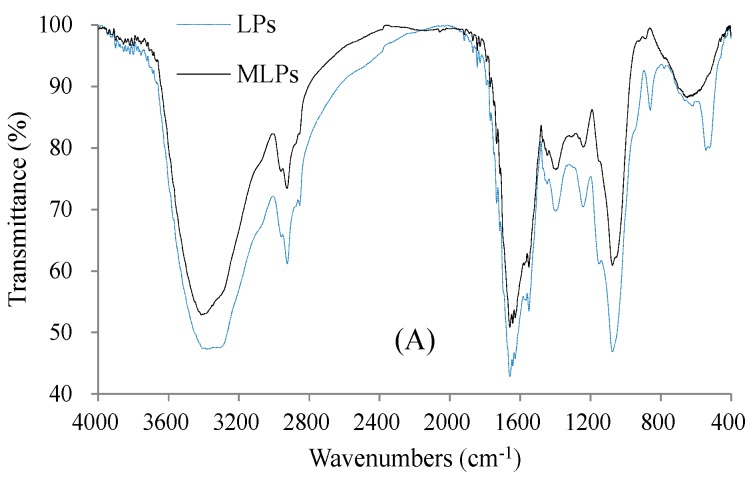

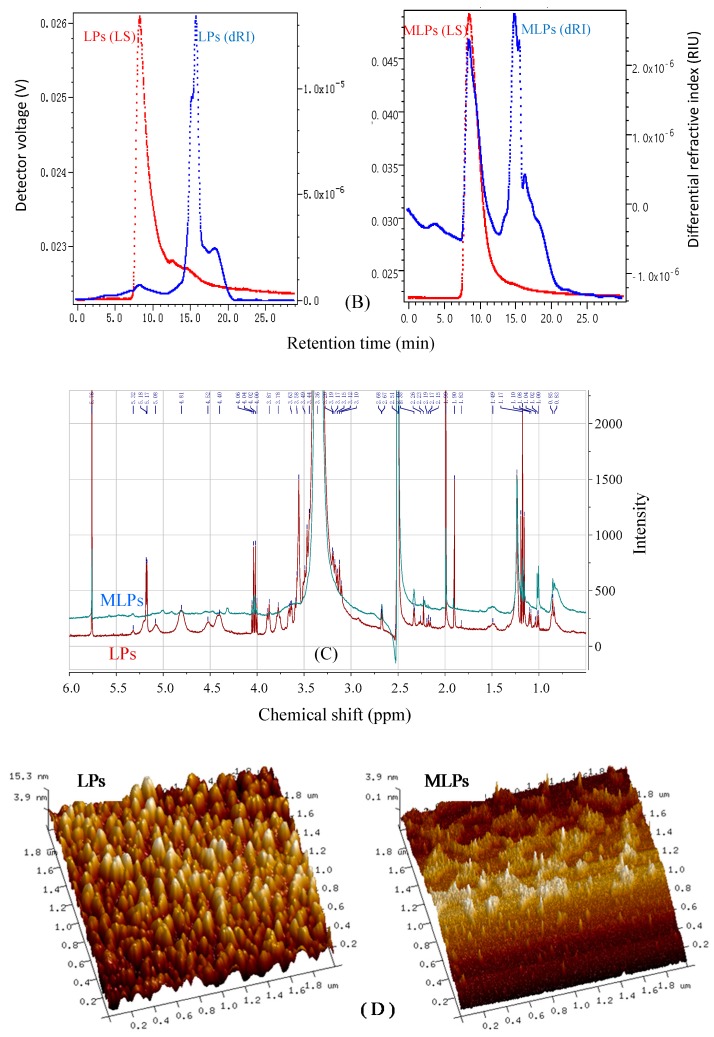
The structural features of LPs and MLPs based on instrumental analyses: (**A**) FTIR spectrum; (**B**) SEC-RI-MALLS chromatogram (RI = refractive index; MALLS = multi-angle laser light scattering); (**C**) 1H NMR spectrum; (**D**) three-dimensional atomic force microscope (AFM) diagram.

The FTIR spectrum of LPs shows the typical characteristics of polysaccharides (Figure 3A), including the stretching vibration of O–H at 3416.11 cm^−1^, the stretching vibration of C–H at 2924.70 cm^−1^, the stretching vibration of C=O at 1668.83 and 1388.04 cm^−1^, and the bending vibration of O–H at 1075.09 cm^−1^. In addition, the bands at 1642.87, 1550.54, and 1241.98 cm^−1^ assigned to the vibration modes of amides indicate the existence of proteins in LPs. The out-of-plane C–H bending vibration of the aromatic ring of LPs contributed to the band at 880.53 cm^−1^ [4], which disappeared in the spectrum of MLPs probably due to the MR-related consumption of aromatic amino acid residues.

The glycosidic linkage locations of the polysaccharides were preliminarily determined by the consumption of periodate and the production of formic acid in the periodate oxidation reaction. LPs accounted for the periodate consumption of 1.92 mmol/g and the formic acid production of 0.40 mmol/g, and those of MLPs were 1.56 mmol/g and 0.48 mmol/g, respectively. The amount ratio of periodate consumption to formic acid production was more than two. It was suggested that many linkages may be of the pyran 1,2-; 1,4-; 1,2,6-; 1,4,6-; 1,2,4- linked forms or the furan 1-linked forms [15]. Moreover, the relatively higher formic acid production of MLPs suggested that they possess more branched structures compared with LPs.

The signals of the multi-angle laser light scattering (MALLS) and the refractive index (RI) in the HPSEC chromatograms (Figure 3B) indicate the molar mass and concentration of polymers, respectively. The RI chromatograms indicate that the *M_w_* distributions of the LPs and MLPs are significantly different, and the detailed data of the LP and MLP fractions are summarized (Table 2). The molecular weights of the LPs and MLPs do not decrease with increasing retention time, as reported previously, indicating the coexistence of different conformations [16,17]. LPs contain fractions with *M_w_* ranging from 2.13 × 10^3^ Da to 8.72 × 10^5^ Da, in which ≤7.07 kDa fractions account for the mass fraction of 94%. The result of *M_w_* distribution is in accordance with the above chromatograms in Figure 1A and Figure 2D. MLPs are composed of fractions with higher *M_w_* ranging from 2.78 × 10^4^ Da to 1.00 × 10^6^ Da, in which ≥264.1 kDa fractions account for the mass fraction of 45%. 

The detailed information about the proton environment of LPs and MLPs was obtained using ^1^H NMR (Figure 3C). The resonances at 2.50 and 3.36 ppm were due to the DMSO solvent and H_2_O in DMSO, respectively. The signals in the chemical shift (*δ*) range of 5.0–5.5 ppm indicated the existence of *α*-glycosidic anomeric protons, while those in the range of 4.4–5.0 ppm belonged to the *β*-glycosidic counterparts [18]. In addition, other non-anomeric protons appeared in the region of 3.2–4.5 ppm. The six peaks in the anomeric region (*δ* 4.4–5.5 ppm) meant that LPs mainly contained six kinds of sugar residues. The signal at 5.76 ppm was derived from the aromatic protons of amino acid residues [19]. Furthermore, the signals in the range of 0.8–3.2 ppm indicated the presence of *β*–H and *γ*–CH_3_, as well as the -CH_2_ and -CH from amino acid residues [19], in which 1.09–1.19 ppm signals responded to the methyl group of rhamnose [20]; 2.16 and 2.25 ppm signals arose from the proton and carbon of O–acetyl groups [21]; and the 2.624 ppm signal belonged to the proton of the hydroxyl groups [22]. MLPs had significantly weaker signal intensity in the range of 3.2–5.5 ppm compared with LPs. This might be due to the higher *M_w_* of the former which led to a somewhat worse solubility [20], or the consumption of carbonyl groups that impacted on the chemical shift of glycosidic anomeric protons.

The observed atomic force microscope (AFM) shapes (Figure 3D) indicate that LPs existe as circular particles with the diameter range of 20–160 nm and the height up to 15.3 nm, and MLPs exist as cross-linked chains with the chain width about 80 nm and the height up to 3.9 nm. Both of the two shapes of longan pulp polysaccharides have been observed in previous studies [16,17]. The detected heights of LPs and MLPs were considerably larger than that of a glucose unit (about 0.3 nm), indicating the excessive aggregation of macromolecules [23].

### 2.5. In Vitro Activities of LPs and MLPs

**Figure 4:**
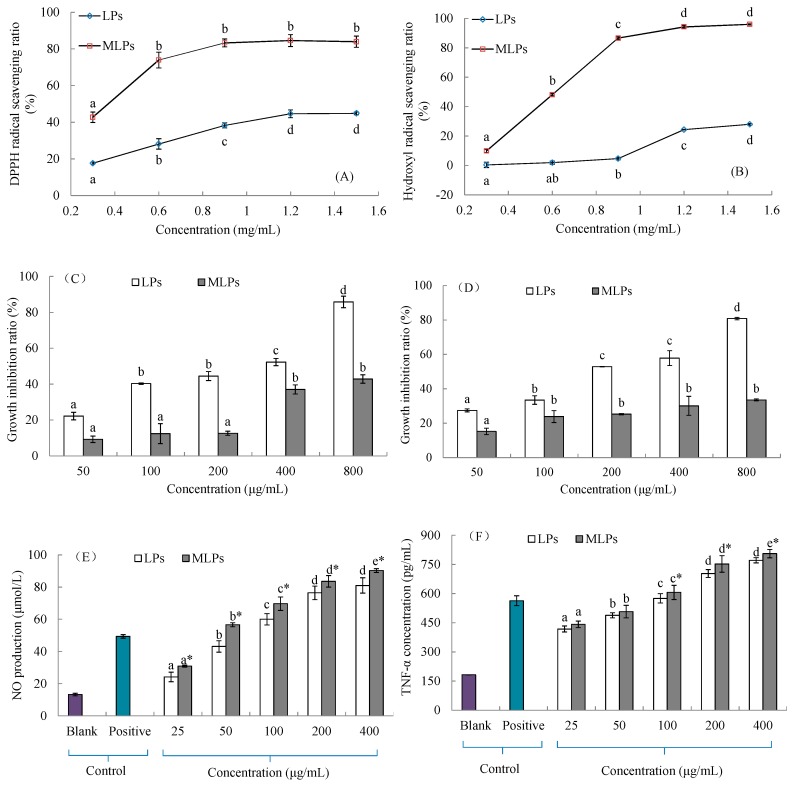
The in vitro activities of LPs and MLPs: (**A**) The scavenging ability against DPPH radicals; (**B**) the scavenging ability against hydroxyl radicals; (**C**) the growth-inhibitory ability against HepG2 cells; (**D**) the growth-inhibitory ability against SGC7901 cells; (**E**) the immunostimulatory effect on macrophage NO production; (**F**) the immunostimulatory effect on macrophage TNF-*α* secretion. The statistical differences at the *p* < 0.05 level among the five concentrations of samples are indicated by different letters, and that between LPs and MLPs at the same concentration is indicated by the symbol ‘*’. Lipopolysaccharide (5 μg/mL) was used as the positive control.

The free radical scavenging ability, as one of the important antioxidant mechanisms of polysaccharides [24], was evaluated to investigate the antioxidant difference between LPs and MLPs. Obviously, MLPs possessed stronger scavenging abilities against DPPH radicals and hydroxyl radicals, as seen in Figure 4. The DPPH radical scavenging ratio of LPs increased steadily from 17.66% at 0.3 mg/mL to 44.60% at 1.2 mg/mL (*p* < 0.05). The ratio of 0.3 mg/mL MLPs was comparable to that of 1.2 mg/mL LPs (*p* < 0.05). The DPPH radical scavenging ratios (about 80%) of MLPs at various concentrations ranged from 0.6 mg/mL to 1.5 mg/mL and do not show significant difference (*p* > 0.05). The hydroxyl radical scavenging ratio of LPs increased slightly from 0.39% at 0.3 mg/mL to 4.67% at 0.9 mg/mL, but then increased obviously to 28.02%. On the contrary, a sharp ratio increase of MLPs was observed in the concentration range of 0.3–0.9 mg/mL, but subsequent increases at higher concentrations were limited. The highest ratio was 95.99% at 1.5 mg/mL, which shows no significant difference from the ratio at 1.2 mg/mL (*p* > 0.05).

Gastric cancer and liver cancer are, respectively, the 2nd and 3rd most common malignancies in East Asia, as well as two of the leading causes of cancer deaths in China [25]. The anticancer activities of LPs and MLPs were evaluated by the growth-inhibitory abilities against HepG2 and SGC7901 cells. As seen in Figure 4C,D, LPs exhibited stronger growth-inhibitory abilities than MLPs at the same concentration in the range of 50–800 μg/mL (*p* < 0.05). The growth-inhibitory effects of LPs were in a concentration-dependent manner, and the highest inhibition ratios against HepG2 and SGC7901 cells were 85.84% and 80.83%, respectively. The concentration-related increase of the HepG2 cell inhibition ratio of MLPs appeared at 400 μg/mL (*p* < 0.05), which showed no significant difference from that at 800 μg/mL (*p* > 0.05). The SGC7901 cell inhibition ratios of MLPs in the range of 100–800 μg/mL did not show significant differences (*p* > 0.05), and the lowest ratio was 15.22% at 50 μg/mL.

NO and TNF-*α* production are two of the important indexes associated with macrophage activation and serve as an adaptive component of innate immunity. Both LPs and MLPs showed significant immunostimulatory effects compared with the blank control, and their effects were in a concentration-dependent manner (Figure 4E,F). LPs exhibited weaker effects on NO production than MLPs at various concentrations in the range of 25–400 μg/mL (*p* < 0.05). Their effects in the range of 100–400 μg/mL were comparable to the positive control (5 μg/mL lipopolysaccharide) (*p* < 0.05). The highest NO production values of LPs and MLPs observed at 400 μg/mL were 81.05 μmol/L and 91.32 μmol/L, respectively. Moreover, LPs also showed weaker immunostimulatory effects on TNF-*α* secretion than MLPs in the range of 100–400 μg/mL (*p* < 0.05). The highest TNF-*α* secretion values of LPs and MLPs observed at 400 μg/mL were, respectively, 771.29 μmol/L and 805.92 μmol/L, which were significantly higher than that of lipopolysaccharide (563.46 μmol/L) (*p* < 0.05).

## 3. Discussion

### 3.1. Effects of Lys-Involved MR on the Structural Features of LPs

MR is a natural and nontoxic method for modifying polysaccharides, and the related modifications mainly focus on the covalent cross-linking with proteins or peptides [11]. Amino acids, by contrast, have been rarely used for the MR-based modification of polysaccharides [12]. Lys, which is quite sensitive to the MR with various carbohydrates [26], has been finally chosen to modify LPs. According to the reaction performance and the structural comparison between LPs and MLPs, it is suggested that the effects of Lys-involved MR on the structure of LPs were related to three aspects: (1) the conformational dissociation of LPs; (2) the MR between LPs and Lys; and (3) the self-aggregation of Lys-grafted MLPs.

The 1.40 × 10^4^ g/mol fraction of longan pulp polysaccharides aggregated as sphere-like particles in distilled water. The molecular aggregation, commonly related to the branched and entangled structure of polysaccharides [27], might be disassociated under the alkaline conditions of MR [17]. Meanwhile, the high reaction temperature could also break the intermolecular and intramolecular forces that are responsible for maintaining the compact conformation of LPs. To some extent, the depolymerization is irreversible and is beneficial for the MR with Lys. 

About 12% of free amino groups in the LPs–Lys MR system were consumed for the preparation of MLPs (data not shown). However, the content of grafted Lys in MLPs was close to 25.28 g/100 g DW (Table 1). It is suggested that most of Lys molecules devoted one of their free amino groups to the MR under the condition that free amino groups were much more abundant relative to carbonyls. In other words, Lys did not contribute to the intermolecular and intramolecular linkage of LPs during the MR. Therefore, the molecular weight of LPs was not significantly increased by the reaction, as seen in Figure 2C. It has been reported that the α-amino group is a major glycated site and the ε-amino group plays a predominant role in cross-linking [28]. In view of that, it can be concluded that the MR occurred between the carbonyl groups of LPs and the α-amino groups of Lys.

Paradoxically, MLPs obviously show a lower number of free amino groups and a larger *M_w_* range compared with LPs (Table 1 and Table 2). The molecules of MLPs in the alkaline environment possessed a lot of negative charges, which prevented the molecular aggregation by electrostatic repulsion. However, the subsequent dialysis changed the environment to neutral. As a result, the intermolecular interaction of MLPs formed network-like aggregates by hydrogen-bond interactions and electrostatic attractions [9], as observed in Figure 3D.

### 3.2. Effects of Lysine-Involved MR on the In Vitro Activities of LPs

The polysaccharide conjugates prepared by MR, which have fewer safety issues than chemically modified polysaccharides, show various functional properties, such as antioxidant, anticarcinogenic, and immunomodulatory activities [7,8,10,29]. However, the effect of MR on the properties of polysaccharides has been rarely studied. In the present work, the MR-based modification shows different effects on the in vitro activities of LPs. As LPs–Lys conjugates, MLPs possess stronger free radical scavenging abilities and immunostimulating effects, but weaker growth-inhibitory activities against cancer cells, compared with LPs (Figure 4).

In the advance stage of MR, the degradation of Amadori compounds at pH values above 7 involves mainly 2,3-enolization, with reductones being formed [10]. The reductones can react with free radicals to stabilize and terminate free radical chain reactions by donating electrons. In addition, the nitrogenous polymers and co-polymers of brown coloration, known as melanoidins and formed in the final stage [10], have the excellent capacities of free radical scavenging and metal chelating [30]. It is suggested that the relatively stronger free radical scavenging abilities of MLPs might be primarily related to the formation of reductones and melanoidins.

The underlying causes involved in the weakened inhibition effects of MLPs on cancer cells are complex. The decreased content of free amino groups, associated with the decrease of positive charges, might weaken the electrostatic interaction between MLPs and negatively-charged cancer cell membranes for triggering certain signal pathways, which leads to the growth inhibition of cancer cells. The mannose contents of 39 lotus root polysaccharides showed a significantly positive correlation with their HepG2 cell inhibitory activities. The result implied the potential relationship between the branched mannose residues of polysaccharides and the highly-expressed mannose receptors of HepG2 cells [31]. In addition, the *α*-L-Rha*p*-(1→ of polysaccharides significantly contributes to their growth-inhibitory effects against cancer cells [6]. The mannose and rhamnose of LPs might conjugate with Lys via MR. Accordingly, MLPs have weakened affinities with cancer cells, as well as growth inhibitory effects. 

Toll-like receptor 4 (TLR-4) has been confirmed to be the main receptor delivering the primary signals of longan polysaccharides to active macrophages [3]. Most of the reported polysaccharide ligands of TLR-4 are in the *M_w_* range of 10–1000 kDa, suggesting that its complex structure is difficult to discern at <10 kDa polysaccharides [5]. Compared with LPs, MLPs may contain more repetitive structures responsible for cross-linking receptors or other membrane targets in a multivalent manner to enhance immunostimulatory effects [32]. Moreover, the network-like conformation of MLPs may be more suitable for recognition by the extracellular and horseshoe-shaped domains of TLR-4, compared with the particle-like conformation of LPs. The grafted Lys molecules may enhance the interaction between MLPs and receptors in view of the protein–protein interactions being significantly stronger than polysaccharide–protein binding affinities [5].

## 4. Materials and Methods

### 4.1. Materials and Cells

Fresh longan fruits (*Shixia* cultivar) were harvested on 10 July 2017 from Yueyunshan Agriculture Development Co., Ltd. (Maoming, China). RAW264.7 macrophages, HepG2 human hepatoma cells, and SGC7901 human gastric cancer cells were purchased from Biosea Biotechnology Co., Ltd. (Wuhan, China).

### 4.2. Extraction and Isolation of Longan Pulp Polysaccharides

Polysaccharides were extracted from longan pulps according to our previous method [4] with slight modifications. In brief, 1.0 kg pulp was homogenized (10,000 r/min, 10 min) in 1.5 L distilled water, followed by 120 r/min stirring for 3 h at pH 8.0 and room temperature. The extracted solution isolated via centrifugation (3396× *g*, 10 min) was adjusted to pH 5.0 and kept at 4 °C for 3 h to precipitate proteins. The resulting supernatant was vacuum-concentrated at 70 °C to about 250 mL and then mixed with 95% ethanol (750 mL) at 4 °C for 12 h to precipitate polysaccharides, which were lyophilized for the next purification. 

Fifty milligrams of the polysaccharides were dissolved in 10 mL distilled water. The polysaccharide solution was pretreated with centrifugation (11,180× *g*, 10 min) and filtration through a 0.22 μm filter for injection onto a HiPrep Sephacryl S-100 gel column (26 × 60 cm, GE Healthcare, Buckinghamshire, UK). The gel filtration chromatography was performed on a 1500 SSI Series System mainly composed of a binary pump and an ultraviolet detector (Scientific Systems, Inc., State College, PA, USA). The detection wavelength of the detector was 280 nm. Phosphate buffer (0.05 mol/L, pH 7.2) was used as the mobile phase at a flow rate of 1.3 mL/min. Eluent was collected in tubes at 6 min intervals. According to the elution profile determined by the phenol-sulfuric acid method [33], the main polysaccharide fractions were defined and dialyzed (MWCO: 3,500 Da, Spectrum Laboratories, CA, USA) against distilled water at 4 °C for 72 h. The factions were finally obtained by lyophilization and named as LPs. 

### 4.3. MR between LPs and Amino Acids

LPs and amino acids (glycine, Gly; L-lysine, Lys; L-proline, Pro) were dissolved together in 100 mL NaOH solution (pH 9.0) to the final concentration of 2.5 mg/mL. Ten milliliters of the solution were added into a 25 mL penicillin bottle and sealed. The bottles were placed in a 100 °C water bath for 1–6 h, and then transferred to an ice-water bath to end the MR between LPs and amino acids. The browning degree was determined by the 420 nm absorbance; the *M_w_* distribution was analyzed by a high-performance size exclusion chromatography (HPSEC) method [4]. 

### 4.4. Preparation of MR-Modified LPs

According to the procedure described above, the LPs–Lys MR lasted for 4 h. The reaction mixture was dialyzed (MWCO: 500 Da, Spectrum Laboratories) against distilled water at 4 °C for 72 h, and then lyophilized to obtain the MR-modified LPs (MLPs).

### 4.5. Composition Analysis

The polysaccharide contents of LPs and MLPs, which were expressed as glucose equivalents contained in a 100 g sample (dry weight, DW), were measured by the phenol-sulfuric acid method [33]. Their protein contents (g albumin equivalents/100g DW) were determined using a Coomassie brilliant blue staining-based protein determination kit (Nanjing Jiancheng Bioengineering Institute, Nanjing, China), and their free amino group contents were measured by the modified OPA method of Xue et al. [34] and expressed as lysine equivalents (μmol/g DW). In addition, the monosaccharide composition was analyzed by a reversed-phase high-performance liquid chromatography (HPLC) method with 1-phenyl-3-methyl-5-pyrazolone-based precolumn derivatization [35].

### 4.6. Structural Analysis

The ultraviolet–visible (UV–Vis) spectra and Fourier transform infrared (FTIR) spectra of LPs and MLPs were measured by the methods of Han et al. [4]; periodate oxidation analyses were performed with the method described by Hu et al. [15]; and atomic force microscope (AFM) images were observed according to our previous method [17].

The *M_w_* distributions of LPs and MLPs were detected using a HPSEC system mainly composed of a model 510 pump (Waters, Milford, MA, USA), a PL aquagel-OH 40 column (8 μm, 7.5 × 300 mm, Agilent), a multi-angle laser light scattering (MALLS) detector (DAWN HELEOS-II 18, Wyatt Technology Co., Santa Barbara, CA, USA), and a differential refractive index (RI) detector (Optilab rEX, Wyatt Technology Co., Santa Barbara, CA, USA). Ammonium acetate solution (0.2 mol/L) was used as the mobile phase at a flow rate of 0.7 mL/min. Five milligrams of samples were dissolved in 2 mL ammonium acetate solution, followed by filtration through a 0.22 μm filter for injection. The injection volume was 20 μL and the column temperature was 30 °C.

After having been kept in a vacuum drier for a week, samples (30–40 mg) were put into a 5 mm NMR tube and dissolved in 1.0 mL dimethyl sulfoxide (DMSO). A Bruker AM 400 MHz spectrometer (Bruker, Rheinstetten, Germany) was used to record ^1^H NMR spectrum with the operating frequency of 400.13 MHz at 30 °C.

### 4.7. Evaluation of In Vitro Activities

The antioxidant activities of samples were evaluated by DPPH radical scavenging ability and hydroxyl radical scavenging ability, which were respectively measured by the methods of Brand-Williams et al. [36] and Xiong et al. [37]. The methods were both slightly modified. Tubes containing 1.5 mL sample aqueous solution and 0.5 mL DPPH alcohol solution (0.2 mol/mL) were dark-incubated at room temperature for 30 min to measure the 517 nm absorbance. In addition, 1.0 mL sample aqueous solution incorporated with 1.0 mL FeSO_4_ solution (1.5 mmol/L), 0.6 mL H_2_O_2_ (6.0 mmol/L) and 0.4 mL salicylic acid (2.0 mmol/L) was incubated in a 37 °C water bath for 1 h to measure the 510 nm absorbance. The final sample concentration in the reaction systems ranged from 0.3 mg/mL to 1.5 mg/mL, and the corresponding radical scavenging ratio (%) was calculated.

The growth-inhibitory abilities of samples against HepG2 cells and SGC7901 cells in the logarithmic phase was determined by the method of Han et al. [4], using a cell counting kit (CCK-8, EnoGene Biotech Co., Ltd., Nanjin, China). The inhibition ratio (%) of sample in the concentration range of 50–800 μg/mL was calculated.

The immunostimulating effects of samples on the NO production and tumor necrosis factor α (TNF-*α*) secretion of RAW264.7 macrophages were evaluated by our previous method [3]. The NO concentration (μmol/L) in the cell culture medium was measured by the Griess method and expressed as sodium nitrite equivalents, and the TNF-*α* concentration (pg/mL) was determined using a mouse TNF-*α* ELISA kit (Neobioscience Technology Co., Shenzhen, China).

### 4.8. Statistical Analysis

Data were expressed as means ± standard deviations (n = 3–6). The statistically significant difference (*p* < 0.05) between groups was analyzed by one-way analysis of variance with the Student–Newman–Keuls test using the IBM SPSS Statistics 19 software (IBM, Armonk, NY, USA). 

## 5. Conclusions

LPs, which were extracted from longan pulps and isolated by gel filtration chromatography, are the heteropolysaccharides mainly composed of Glc, Gal, Man, Rha, GlcA, Rib, and GalA at a molar ratio of 1.00:0.21:0.16:0.15:0.12:0.05:0.04. Their molecular weights mostly range from 2.13 kDa to 7.07 kDa. Lys shows a relatively better performance to the MR with LPs than Pro and Gly, according to the changes of the reaction system in browning degree and *M_w_* distribution. The formation of MLPs via LPs–Lys MR may involve three important aspects. Firstly, the compact conformation of LPs dissociates under an alkaline environment and high temperature. Secondly, Lys molecules conjugate with the carbonyls of LPs mostly by one of their amino groups. Thirdly, the conjugated Lys molecules do not obviously increase the *M_w_* of LPs, but result in the subsequent self-aggregation through hydrogen-bond interactions and electrostatic attractions under a neutral environment to form network-like conformation. Compared with LPs, MLPs show relatively stronger free radical scavenging abilities and immunostimulatory effects on macrophages, but weaker growth-inhibitory abilities against cancer cells. The changes, associated with the Lys-based modification, are related to complicated structure–activity relationships. The present work indicates that the amino acid-involved MR is an effective strategy for the structural modification of polysaccharides. The reaction not only contributes to the changes in their primary structures, but also triggers a series of changes in their advanced structures. These changes mean great challenges for the expected improvement on the functional properties of polysaccharides.

## Figures and Tables

**Table 1:** The chemical composition of LPs and MLPs.

Composition		LPs	MLPs
Polysaccharide content (g/100 g DW) *		90.11 ± 0.15	66.08 ± 0.56
Protein content (g/100 g DW)		6.54 ± 0.17	5.29 ± 0.10
Free amino group content (μmol/g DW)		25.92 ± 0.14	16.35 ± 0.44
Molar ratio of monosaccharide composition	Mannose	0.16	0.19
	Ribose	0.05	0.01
	Rhamnose	0.15	0.17
	Glucuronic acid	0.12	0.13
	Galacturonic acid	0.04	0.03
	Glucose	1.00	1.00
	Galactose	0.21	0.24

* The contents were expressed as the corresponding equivalents contained in the sample (dry weight, DW).

**Table 2:** The weight-average molecular weight distribution of LPs and MLPs.

Sample	Retention Time (min)	Weight-Average Molecular Weight (Da)	Mass Fraction (%)
LPs	7.216–11.947	8.716 × 10^5^ (±0.863%)	3.6
	12.005–13.833	1.174 × 10^5^ (±1.593%)	2.4
	13.862–15.343	7.068 × 10^3^ (±2.736%)	24.6
	15.372–16.590	2.130 × 10^3^ (±4.084%)	43.0
	16.619–17.432	5.678 × 10^3^ (±4.028%)	7.7
	17.461–20.131	4.529 × 10^3^ (±4.060%)	18.7
MLPs	7.297–9.288	1.004 × 10^6^ (±0.441%)	23.0
	9.349–12.637	6.861 × 10^5^ (±0.414%)	18.6
	12.667–13.692	2.641 × 10^5^ (±0.565%)	3.4
	13.692–15.261	4.056 × 10^4^ (±0.867%)	24.0
	15.291–15.925	2.782 × 10^4^ (±1.096%)	10.0
	15.955–16.920	4.337 × 10^4^ (±0.984%)	7.6
	16.950–17.674	4.231 × 10^4^ (±0.908%)	4.5
	17.705–20.842	4.958 × 10^4^ (±1.361%)	8.9
